# Umbilical cord tissue cryopreservation: a short review

**DOI:** 10.1186/s13287-018-0992-0

**Published:** 2018-09-15

**Authors:** Irina Arutyunyan, Timur Fatkhudinov, Gennady Sukhikh

**Affiliations:** 1 National Medical Research Center for Obstetrics, Gynecology and Perinatology named after Academician V.I. Kulakov of Ministry of Healthcare of Russian Federation, Moscow, Russia; 2 Peoples’ Friendship University of Russia, Moscow, Russia

**Keywords:** Umbilical cord, Tissue cryopreservation, Cryoprotectants, Vessels, Graft, Mesenchymal stem cells

## Abstract

In this review we present current evidence on the possibility of umbilical cord tissue cryopreservation for subsequent clinical use. Protocols for obtaining umbilical cord-derived vessels, Wharton’s jelly-based grafts, multipotent stromal cells, and other biomedical products from cryopreserved umbilical cords are highlighted, and their prospective clinical applications are discussed. Examination of recent literature indicates we should expect high demand for cryopreservation of umbilical cord tissues in the near future.

## Background

In 1974, umbilical cord blood (UCB) was reported to be a source of hematopoietic stem and progenitor cells [1], and in 1988, the first transplantation of cryopreserved UCB to an infant with Fanconi anemia, an inherited bone marrow disease, was performed in France [2]. Over the next 30 years, numerous studies were published demonstrating the regenerative potential of particular UCB-derived cell populations, and a global network of public and private biobanks of UCB was established [3, 4].

For many years, the solid tissues of umbilical cord (UC) were treated as a valueless medical waste. The past decade, however, has been notable for intensive development of biomedical products on the basis of UC tissues—for example, UC-derived mesenchymal stem cells (MSCs), which can be obtained from total UC or its dissected compartments (perivascular, intervascular, subamniotic zones of Wharton’s jelly, and subendothelial layer of blood vessels). With their high proliferative potential, karyotype and phenotype stability, differentiation plasticity, paracrine activity, and immunomodulatory properties, UC-derived MSCs can claim the title of the new “gold standard”, ousting the renowned bone marrow-derived MSCs [5–7]. Other examples of UC-derived biomedical products are decellularized UC vessels used as grafts for vascular surgery [8–10] and Wharton’s jelly-derived extracellular matrix for wound healing [11].

The main disadvantage of UC as a tissue source is its transientness: it is only available during a short time period immediately postpartum. An effective solution to this problem may be provided by its careful cryopreservation with all efforts made to protect the useful components (cells, stromal matrices, specialized tissues) during storage. This short review presents current evidence on the possibilities of UC tissue cryopreservation, which would allow the use of its particular components in cell therapy and regenerative medicine.

## Cryopreservation of UC-derived vessels

Surgical reconstruction of small vessels involves autologous transplantation as a gold standard, but this is not always affordable [9]. Decellularized umbilical vessels of appropriate diameter and considerable length without branches represent a suitable material for vascular prostheses [8–10, 12–15]. This makes the effective cryopreservation of UC-derived vessels highly important for vascular surgery. Experiments show that although cryopreservation of the UC vessels significantly affects the subsequent decellularization efficacy (which may be attributed to condensation of extracellular matrix during freezing), it has no influence on their mechanical properties such as stiffness, burst pressure, and suture retention strength [12].

The protocols for cryopreservation of UC blood vessels as a biomaterial for allogeneic transplantation do not imply the preservation of the cellular component. For this reason, the cryopreservation medium is composed of saline with no cryoprotectants. It is used in > 20 volume excess to the volume of fresh material at a cooling rate of 1 °C/min, with subsequent storage at − 20°С [12]. In the case of cryopreservation of UC blood vessels for autologous transplantation, preservation of the living cells in the blood vessel walls would make sense. However, no appropriate investigation of cell survival during UC vessel cryostorage with cryoprotectants has been done [15]. At the same time, the possible utility of the UC blood vessel-derived scaffolds is not confined to vascular surgery, but may be extended to tissue engineering options for nerve [16], periodontal tissue [17], and musculoskeletal soft tissue [18] regeneration.

## Cryopreservation of Wharton’s jelly

UC stroma contains a unique gelatinous substance which is missing in the human body after birth. It is called Wharton’s jelly (WJ) after Thomas Wharton (1614–1673), an English physician and anatomist. WJ protects the blood vessels (two umbilical arteries and one umbilical vein) from clumping and also ensures cord flexibility. It is a rich reservoir of growth factors and contains significant amounts of extracellular matrix components such as collagen (types I, III, IV, and V), hyaluronic acid, and several sulfated glycosaminoglycans [5]. Such an attractive combination of biomechanical and biochemical features makes WJ an important candidate material for medical applications. For example, a biomimetic spongy scaffold, which had been produced from decellularized WJ by using a freeze-drying technique, was shown to improve the attachment, penetration, and growth of fibroblasts and speed up wound healing processes [11].

Decellularized allografts are regularly used in clinical practice, particularly in ophthalmology and wound treatment [19]. One of the most common allografts is based on the amniotic membrane, which consists of a monolayer of simple epithelium with a thick basement membrane and the underlying avascular stromal region. This graft is obtained by dehydration or, alternatively, by freezing, which better protects the tissue architecture and the biologically active molecules of the extracellular matrix [19]. Allograft material of similar structure on the basis of WJ from cryopreserved UCs was introduced in 2014. The content of high molecular weight hyaluronic acid (which is suggested to be the key isoform of hyaluronic acid responsible for the therapeutic properties) after thawing is reportedly higher in WJ than in the amniotic membrane. Moreover, extracts of UC tissue, but not amniotic membrane, promote anti-inflammatory cytokine IL-10 expression and a decrease in pro-inflammatory cytokine IL-12 expression in the macrophage cell line RAW264.7. This result indicates that the UC allografts have certain advantages [19].

Similar to the case of vascular tissue, cryopreservation of UC for obtaining the WJ matrices does not imply the preservation of the cellular component. For this reason, the fresh tissue is simply cooled to − 80 °С without cryoprotectants [19]. Grafts obtained on the basis of cryopreserved WJ have already proven to be effective in the treatment of spina bifida [20], complex lower extremity ulcers with exposed bone, tendon, muscle, and/or joint capsule, as well as multiple comorbidities including diabetes, ischemia, and underlying osteomyelitis [21–23].

## Cryopreservation of the UC cellular component

Initiation of pregnancy with sperm that had been stored on dry ice for a short while was first done in 1953. The subsequent introduction of liquid nitrogen for the long-term cryostorage of sperm in the early 1960s substantially contributed to the efficacy of the approach [24]. Contemporary cryotechnologies allow the long-term preservation of cells both in suspensions and within whole tissue fragments (e.g., whole adipose tissue [25, 26], dental follicle tissue [27], bone marrow fragments [28], testicular [29] and ovarian [30] tissues), from which cells can be successfully isolated after thawing. Compared with the storage of isolated cells, the storage of unprocessed tissues has a number of advantages: minimization of time, labor, and material expenses; storage of cells in their natural environments; future possibilities of cell isolation and expansion in accordance with as yet unknown future standards.

Full-scale experimental studies of UC tissue cryopreservation started about 10 years ago. Several types of UC cells, including epithelial and endothelial cells, are valuable for regenerative medicine and tissue engineering and can be cultured [31–33]. Quite recently, an effective method for human umbilical vein endothelial cell (HUVEC) cryopreservation was reported [34]; importantly, the stage of cell culturing and expansion before transfer of the samples to the biobank is omitted in this procedure. Briefly, primary endothelium pellets, which are isolated from UC by enzymatic digestion, are frozen and placed in a liquid nitrogen freezer for long-term storage followed by fast thawing at + 37 °C. With this protocol, 14 viable HUVEC cultures have been successfully obtained from 17 primary endothelial pellets, which is an 82% success rate. The authors consider this approach helpful in improving the efficiency and logistics of biobanking, especially when processing large collections of endothelial samples [34].

However, the majority of such studies are predominantly focused on the isolation of MSCs from cryopreserved UC tissue. It is important to note that it is hypothesized that the therapeutic potential of MSCs substantially reduces during cryostorage (the so-called “cryo stun effect”), which explains the multiple failures of clinical trials utilizing cell transplants immediately after thawing [35]. In this regard, cryopreservation of whole UC tissue for subsequent isolation and expansion of MSCs for experimental or clinical purposes represents a strategy of choice.

The pioneering studies in this field were unsuccessful as no MSC cultures were obtained from WJ samples cryopreserved for 1 week, 1 month, or 6 months in liquid nitrogen, despite cryoprotection with 10% dimethyl sulfoxide (DMSO) and 5% glycerol [36]. Both DMSO and glycerol are renowned cryoprotectants used to prevent cell damage during freezing of cell culture stocks by interrupting the intracellular formation of ice crystals. Nevertheless, preservation of living cells in a WJ sample prepared with 1.5 M DMSO and 0.1 М sucrose by slow freezing (but not by vitrification) was demonstrated in 2012 [37]. Even more convincing data on obtaining MSCs from UC tissues after storage in liquid nitrogen were published in 2013; these MSCs were phenotypically and functionally identical to those obtained from fresh tissues [38]. Several scientific groups have reported their success throughout 2014–2018, suggesting a variety of protocols for the cryopreservation of UC tissue. The results are presented in Table 1.

**Table 1:** Results of experimental studies on obtaining MSCs from cryopreserved UC tissues

Cryopreservation protocol (cryoprotectant; freezing; storage; thawing)	Method used for obtaining primary cultures	First migratory cells from tissue fragments or first cell colonies	MSC phenotype	Differentiating potential of MSCs (in vitro)	Other findings	Reference
10% DMSO; controlled freezing to − 180 °С; storage in liquid nitrogen for 5 years; thawing at room temperature for 30 s, followed by a complete thaw in a 37 °C water bath	Explants	After 10–14 days of culture	CD44+, CD90+, CD105+, CD34^−^, CD45^−^	Adipogenic, osteogenic	MSCs isolated from thawed tissue displayed lower plating efficiency, along with a prolonged cell doubling time and fewer total cell doublings, compared with MSCs from fresh tissue	[47]
10% DMSO + 0.2/0.5 М sucrose; freezing to − 80 °С (1 °C/min); storage in liquid nitrogen for 5–29 days; thawing in a 37 °C water bath	Explants	–	CD73+, CD90+, CD34^−^	Adipogenic, chondrogenic	It took longer to obtain MSCs from cryopreserved UC explants compared with the corresponding fresh explants from the same donor	[43]
					Cryostorage time influenced neither the terms of MSC outgrowth nor their mean population doubling time	
10% DMSO; programmed freezing to − 90 °С; storage in liquid nitrogen for 2–3 months; thawing in a 37 °C water bath	Explants pre-incubated in collagenase IV solution for 30–45 min on ice and covered with a stainless steel mesh to protect the tissue from floating	After 7–9 days of culture	CD90+, CD105+, CD73+, CD13+, CD29+, CD44+, CD54+, CD117+, CD71+, CD146+, HLA^−^ABC+, CD34^−^, CD45^−^, HLA-DR^−^, CD309/VEGF^−^R2/KDR^−^	Adipogenic, osteogenic	MSC cultures were obtained from all examined tissue samples irrespective of the time of incubation with the cryoprotectant (5–60 min)	[48]
					Proliferative activity of MSCs isolated from fresh and frozen biological material did not differ	
10% DMSO (Cryo Sure-DEX40, 55% w/v DMSO + 5% w/v Dextran); slow freezing to − 90 °С; storage in liquid nitrogen for 1 month; thawing in a 37 °C water bath	Explants	After 10–14 days of culture	CD90+, CD105+, CD34^−^, CD45^−^	Adipogenic, chondrogenic, osteogenic	MSCs from cryopreserved UC explants and corresponding fresh explants from the same donor showed similar adipogenic, chondrogenic, and osteogenic in vitro differentiation capacities regardless of growth media used for their isolation and expansion	[49]
					The time required to reach 60% confluence for the post-thaw cultures was longer	
					No difference in doubling population time was observed for the cells derived from pre-freeze tissues vs post-thaw tissues	
STEM-CELLBANKER (contains DMSO and anhydrous dextrose); freezing to − 80 °С (2 °C/min); storage in liquid nitrogen for 2 weeks; thawing in a 37 °C water bath	Explants covered with a stainless steel mesh to protect the tissue from floating	–	CD73+, CD105+, CD90+, CD44+, HLA-ABC+, CD45^−^, CD34^−^, CD14^−^, CD19^−^, HLA-DR^−^	Adipogenic, chondrogenic	Cells derived from UCs after cryostorage retained their immunosuppressive properties, as assessed by allogeneic mixed lymphocyte reactions, and their potential to differentiate into adipocytes and chondrocytes was comparable with cells derived from fresh UC tissue	[44]
Recovery™ Cell Culture Freezing Medium (contains 10% DMSO); programmed freezing to − 150 °С; storage in liquid nitrogen for 1 month; thawing in a 37 °C water bath	Post-thaw WJ was syringed through an 18 G needle a few times to loosen the gelatinous material and dislodge the stem cells; explants for entire cord segments	After 6 days of culture for cell suspensions, after 12 days of culture for explants	CD29+, CD44+, CD73+, CD90+, CD105+, HLA-ABC+, CD14^−^, CD34^−^, CD45^−^, HLA-DR^−^	Chondrogenic, osteogenic	Freezing of freshly dissected WJ worked better than freezing of entire UC segments in terms of higher MSC viability and proliferation rates, lower numbers of annexin-V-positive and sub-G1 cells, and enhanced osteogenic and chondrogenic in vitro differentiation	[50]
10% DMSO or 0.05 M glucose + 0.05 M sucrose + 1.5 M ethylene glycol cocktail; conventional freezing to − 80 °С (1 °C/min) or programmed freezing to − 140 °С; storage in liquid nitrogen for 3 months; thawing in a 37 °C water bath	Enzymatic digestion with DPBS containing 1 mg/ml collagenase type I at 37 °C for 15 min	After 5 days of culture	CD73+, CD90+, CD105+, CD34^−^, CD45^−^	Adipogenic, chondrogenic, osteogenic, hepatogenic	A new cocktail cryoprotectant ensured better preservation of WJ tissue than DMSO	[42]
					Proliferation capacities of the cells isolated from fresh and programmed freezing WJ samples were comparable, proliferation capacity of the cells from conventional freezing WJ samples was significantly lower	
					MSCs from the conventional freezing samples showed upregulated expression of pro-apoptotic factors (BAX, p53, and p21) and downregulated expression of anti-apoptotic factor (BCL2), compared with MSCs from the fresh and programmed freezing samples	
10% DMS; freezing to − 80 °С (1 °C/min); storage in liquid nitrogen for 3 days	Explants or “conditioned explants” (cultured for 2 weeks allowing adaptation to the culture conditions prior to freezing)	After 14 days of culture for explants, after 2–8 days of culture for “conditioned explants”	CD73+, CD90+, CD105+, CD14^−^, CD31^−^, CD34^−^, CD45^−^	Adipogenic, chondrogenic, osteogenic	Cryopreserved “conditioned explants” could be repeatedly cryopreserved to show repeated outgrowth MSCs with the same properties for at least four freeze/thaw/explant culture cycles	[51]
					Increasing the number of freeze/thaw/explant culture cycles to 7 and 10 associated with significant decrease in proliferation capacity	
CryoStor CS10 (contains 10% DMSO); freezing to − 80 °С; storage in liquid nitrogen for 1 month; rapid thawing at 37 °C	Explants (tissue pieces were allowed to rest without medium for 10 min to ensure adherence to the culture plate)	After 14 days of culture	CD73+, CD90+, CD105+, CD34^−^, CD45^−^, CD14^−^, CD19^−^, HLA-DR^−^	–	Two newly developed approaches for quantitative evaluation of UC tissue as a cell source were suggested (a standardized explant approach and a metabolic activity assay)	[52]

As can be seen from Table 1, the majority of effective protocols utilize DMSO as the main cryoprotectant. With its small molecular weight of 78.13 g/mol, DMSO is capable of penetrating into the cell via the plasma membrane, preventing the formation of ice crystals by stable hydrogen bonding with water molecules. DMSO has been successfully used for the cryopreservation of cell cultures since 1959; however, it is now being replaced with DMSO-free standards for cryopreservation media. This is primarily because of the rather high toxicity of DMSO for both the cells and their recipients. DMSO is toxic at temperatures above 4 °С even at low concentrations (about 1% is enough), and with an increase in temperature it quickly decomposes into a mixture of toxic products with the distinctive odor of dimethyl sulfide. Moreover, it is impossible to remove it by washing, even with the use of a specialized system for washing cell transplants. The reasonable alternative is a medium supplemented with a cocktail of non-penetrating cryoprotectants (e.g., glucose, sucrose, galactose, or trehalose) and intracellular cryoprotectants (e.g., ethylene glycol, propylene glycol (1,2-propanediol), glycerol, formamide, methanol, and butanediol) [39–41]. According to pilot studies, this cocktail provides better tissue preservation than DMSO [41, 42]. Product lines of the leading biotech companies are now complemented with DMSO-free cryopreservation media, e.g., CryoSOfree™ DMSO-free Cryopreservation Medium by Merck, STEM-CELLBANKER® DMSO Free - GMP grade by AMSBIO, and ReproCryo DMSO Free Cryopreservation Medium by Stemgent.

Another problem which critically limits the clinical applicability of cryopreserved UC tissue-derived MSCs is the presence of xeno proteins in the cryopreservation medium, which usually contains up to 90% by volume of fetal calf serum. The use of xeno-free media significantly increases the efficacy of MCS isolation from cryopreserved UC samples [43, 44], whereas subsequent growing of the cells in xeno-free conditions facilitates a substantial all-round improvement in their properties (reduced apoptosis and immunogenicity, enhanced proliferation, increased secretion of hepatocyte growth factor and prostaglandin Е2) [45, 46]. It is plausible that the proposed replacement of calf serum with autologous serum or suitable pharmacological substances (e.g., human serum albumin) will eventually prevail. In our opinion, the xeno-free standard for UC tissue cryopreservation should be introduced as soon as possible.

## Current prospects of UC tissue banking

Transplantation of UC-derived MSCs is a subject of increasing interest. More than a hundred clinical trials have been currently announced by the FDA (http://www.clinicaltrials.gov/). UC-derived MSCs are intended for the treatment of cardiovascular, liver, and skeletal muscle failures, autoimmune and neurological disorders, and many other diseases [5–7]. In addition, several clinical trials of WJ-based allografts obtained from cryopreserved material (e.g., NEOX®CORD 1 K by AMNIOX Medical, Inc.), sponsored by biotech companies, are currently in progress. It is of no surprise, therefore, that numerous cryobanks, previously engaged in UC blood storage, now offer UC tissue cryopreservation and storage services. The first ten search results on the query *umbilical cord tissue cryobanking* include five banks located in the USA (Cryo-Cell, ViaCord, CariCord, AlphaCord, and New England Cord Blood Bank), two in the UK (Cells4Life and Future Health Biobank), two in Australia (CellCare and CryoSite), and one in South Africa (Cryo-Save). Whole UC tissue preservation has such important advantages as the total in situ preservation of all cell types and the relatively low costs of the procedure (about 2.5 times lower than the costs of UC blood cryopreservation). In our opinion, the optimal solution can be provided by banking of UC blood as a source of hematopoietic stem cells simultaneously with UC tissues as a source of autologous grafts or neonatal MSCs for autologous transplantation.

## Conclusions

Examination of recent literature indicates we should expect high demand for cryopreservation of human UC tissues in the near future. The choice of a protocol for cryopreservation depends on the task—preservation of blood vessels, WJ, or the cellular component. The efficacy of obtaining living cells from thawed UC tissues is largely influenced by the composition of the cryoprotectant medium, freezing mode, and protocol used for cell isolation. Although few data are available on the survival of endothelial or epithelial cells in cryopreserved UC tissues, some current protocols allow MSCs to be obtained from UC tissues after cryostorage that are phenotypically and functionally identical to those obtained from fresh tissues.

## Abbreviations

DMSO: Dimethyl sulfoxide
MSC: Mesenchymal stem cell
UC: Umbilical cord
UCB: Umbilical cord blood

## References

[1] Knudtzon S (1974). In vitro growth of granulocytic colonies from circulating cells in human cord blood. Blood.

[2] Gluckman E, Broxmeyer HA, Auerbach AD, Friedman HS, Douglas GW, Devergie A, Esperou H, Thierry D, Socie G, Lehn P (1989). Hematopoietic reconstitution in a patient with Fanconi’s anemia by means of umbilical-cord blood from an HLA-identical sibling. N Engl J Med.

[3] Ballen KK, Gluckman E, Broxmeyer HE (2013). Umbilical cord blood transplantation: the first 25 years and beyond. Blood.

[4] Ballen KK, Verter F, Kurtzberg J (2015). Umbilical cord blood donation: public or private?. Bone Marrow Transplant.

[5] Arutyunyan I, Elchaninov A, Makarov A, Fatkhudinov T (2016). Umbilical cord as prospective source for mesenchymal stem cell-based therapy. Stem Cells Int.

[6] Kalaszczynska I, Ferdyn K (2015). Wharton's jelly derived mesenchymal stem cells: future of regenerative medicine? Recent findings and clinical significance. Biomed Res Int.

[7] Ding DC, Chang YH, Shyu WC, Lin SZ (2015). Human umbilical cord mesenchymal stem cells: a new era for stem cell therapy. Cell Transplant.

[8] Mallis P, Gontika I, Poulogiannopoulos T, Zoidakis J, Vlahou A, Michalopoulos E, Chatzistamatiou T, Papassavas A, Stavropoulos-Giokas C (2014). Evaluation of decellularization in umbilical cord artery. Transplant Proc.

[9] Ambler GK, Twine CP (2018). Graft type for femoro-popliteal bypass surgery. Cochrane Database Syst Rev.

[10] Rochon C, Sheiner PA, Sharma J, Rodriguez-Davalos MI, Savino J, Facciuto ME (2013). The utility of recanalized umbilical vein graft to the hepato-pancreato-biliary surgeon. Surg Innov.

[11] Beiki B, Zeynali B, Seyedjafari E (2017). Fabrication of a three dimensional spongy scaffold using human Wharton's jelly derived extra cellular matrix for wound healing. Mater Sci Eng C Mater Biol Appl.

[12] Tuan-Mu HY, Yu CH, Hu JJ (2014). On the decellularization of fresh or frozen human umbilical arteries: implications for small-diameter tissue engineered vascular grafts. Ann Biomed Eng.

[13] Gui L, Muto A, Chan SA, Breuer CK, Niklason LE (2009). Development of decellularized human umbilical arteries as small-diameter vascular grafts. Tissue Eng Part A..

[14] Hoenicka M, Lehle K, Jacobs VR, Schmid FX, Birnbaum DE (2007). Properties of the human umbilical vein as a living scaffold for a tissue-engineered vessel graft. Tissue Eng.

[15] Rodríguez-Rodríguez Victor E., Martínez-González Brenda, Quiroga-Garza Alejandro, Reyes-Hernández Cynthia Guadalupe, de la Fuente-Villarreal David, de la Garza-Castro Oscar, Guzmán-López Santos, Elizondo-Omaña Rodrigo Enrique (2018). Human Umbilical Vessels. ASAIO Journal.

[16] Crouzier T, McClendon T, Tosun Z, McFetridge PS (2009). Inverted human umbilical arteries with tunable wall thicknesses for nerve regeneration. J Biomed Mater Res A.

[17] Goktas S, Pierre N, Abe K, Dmytryk J, McFetridge PS (2010). Cellular interactions and biomechanical properties of a unique vascular-derived scaffold for periodontal tissue regeneration. Tissue Eng Part A.

[18] Abousleiman RI, Reyes Y, McFetridge P, Sikavitsas V (2008). The human umbilical vein: a novel scaffold for musculoskeletal soft tissue regeneration. Artif Organs.

[19] Cooke M, Tan EK, Mandrycky C, He H, O'Connell J, Tseng SC (2014). Comparison of cryopreserved amniotic membrane and umbilical cord tissue with dehydrated amniotic membrane/chorion tissue. J Wound Care.

[20] Papanna R, Fletcher S, Moise KJ, Mann LK, Tseng SC (2016). Cryopreserved human umbilical cord for in utero Myeloschisis repair. Obstet Gynecol.

[21] Raphael A, Gonzales J (2017). Use of cryopreserved umbilical cord with negative pressure wound therapy for complex diabetic ulcers with osteomyelitis. J Wound Care.

[22] Caputo WJ, Vaquero C, Monterosa A, Monterosa P, Johnson E, Beggs D (2016). A retrospective study of cryopreserved umbilical cord as an adjunctive therapy to promote the healing of chronic, complex foot ulcers with underlying osteomyelitis. Wound Repair Regen.

[23] Couture M (2016). A single-center, retrospective study of cryopreserved umbilical cord for wound healing in patients suffering from chronic wounds of the foot and ankle. Wounds.

[24] Rozati H, Handley T, Jayasena CN. Process and pitfalls of sperm cryopreservation. J Clin Med. 2017;6(9) 10.3390/jcm6090089. 10.3390/jcm6090089 PMC5615282 28925939

[25] Choudhery MS, Badowski M, Muise A, Pierce J, Harris DT (2014). Cryopreservation of whole adipose tissue for future use in regenerative medicine. J Surg Res.

[26] Devitt SM, Carter CM, Dierov R, Weiss S, Gersch RP, Percec I (2015). Successful isolation of viable adipose-derived stem cells from human adipose tissue subject to long-term cryopreservation: positive implications for adult stem cell-based therapeutics in patients of advanced age. Stem Cells Int.

[27] Park BW, Jang SJ, Byun JH, Kang YH, Choi MJ, Park WU (2017). Cryopreservation of human dental follicle tissue for use as a resource of autologous mesenchymal stem cells. J Tissue Eng Regen Med.

[28] Carnevale G, Pisciotta A, Riccio M, De Biasi S, Gibellini L, Ferrari A (2016). Optimized cryopreservation and banking of human bone-marrow fragments and stem cells. Biopreserv Biobank.

[29] Unni S, Kasiviswanathan S, D'Souza S, Khavale S, Mukherjee S, Patwardhan S, Bhartiya D (2012). Efficient cryopreservation of testicular tissue: effect of age, sample state, and concentration of cryoprotectant. Fertil Steril.

[30] Fleury A, Pirrello O, Maugard C, Mathelin C, Linck C. Breast cancer and ovarian tissue cryopreservation: review of the literature. J Gynecol Obstet Hum Reprod. 2018;(18):30172–7. 10.1016/j.jogoh.2018.05.008. 10.1016/j.jogoh.2018.05.008 29793036

[31] Saleh R, Reza HM (2017). Short review on human umbilical cord lining epithelial cells and their potential clinical applications. Stem Cell Res Ther.

[32] Tanaka M, Tsuno NH, Fujii T, Todo T, Saito N, Takahashi K (2013). Human umbilical vein endothelial cell vaccine therapy in patients with recurrent glioblastoma. Cancer Sci.

[33] Hayward CJ, Fradette J, Galbraith T, Rémy M, Guignard R, Gauvin R (2013). Harvesting the potential of the human umbilical cord: isolation and characterisation of four cell types for tissue engineering applications. Cells Tissues Organs.

[34] Puzanov MV, Vasilyeva LB, Popova PV, Grineva EN, Dmitrieva RI (2018). New approach to cryopreservation of primary noncultivated human umbilical vein endothelium in biobanking. Biopreserv Biobank.

[35] Moll G, Geißler S, Catar R, Ignatowicz L, Hoogduijn MJ, Strunk D, Bieback K, Ringdén O (2016). Cryopreserved or fresh mesenchymal stromal cells: only a matter of taste or key to unleash the full clinical potential of MSC therapy?. Adv Exp Med Biol.

[36] Chatzistamatiou TK, Papassavas AC, Michalopoulos E, Gamaloutsos C, Mallis P, Gontika I (2014). Optimizing isolation culture and freezing methods to preserve Wharton's jelly's mesenchymal stem cell (MSC) properties: an MSC banking protocol validation for the Hellenic cord blood Bank. Transfusion.

[37] Da-Croce L, Gambarini-Paiva GH, Angelo PC, Bambirra EA, Cabral AC, Godard AL (2013). Comparison of vitrification and slow cooling for umbilical tissues. Cell Tissue Bank.

[38] Choudhery MS, Badowski M, Muise A, Harris DT (2013). Utility of cryopreserved umbilical cord tissue for regenerative medicine. Curr Stem Cell Res Ther.

[39] Best BP (2015). Cryoprotectant toxicity: facts, issues, and questions. Rejuvenation Res.

[40] Rodríguez L, Velasco B, García J, Martín-Henao GA (2005). Evaluation of an automated cell processing device to reduce the dimethyl sulfoxide from hematopoietic grafts after thawing. Transfusion.

[41] Du T, Chao L, Zhao S, Chi L, Li D, Shen Y (2015). Successful cryopreservation of whole sheep ovary by using DMSO-free cryoprotectant. J Assist Reprod Genet.

[42] Shivakumar SB, Bharti D, Subbarao RB, Jang SJ, Park JS, Ullah I (2016). DMSO- and serum-free cryopreservation of Wharton's jelly tissue isolated from human umbilical cord. J Cell Biochem.

[43] Roy S, Arora S, Kumari P, Ta M (2014). A simple and serum-free protocol for cryopreservation of human umbilical cord as source of Wharton's jelly mesenchymal stem cells. Cryobiology.

[44] Shimazu T, Mori Y, Takahashi A, Tsunoda H, Tojo A, Nagamura-Inoue T (2015). Serum- and xeno-free cryopreservation of human umbilical cord tissue as mesenchymal stromal cell source. Cytotherapy.

[45] Hartmann I, Hollweck T, Haffner S, Krebs M, Meiser B, Reichart B (2010). Umbilical cord tissue-derived mesenchymal stem cells grow best under GMP-compliant culture conditions and maintain their phenotypic and functional properties. J Immunol Methods.

[46] Swamynathan P, Venugopal P, Kannan S, Thej C, Kolkundar U, Bhagwat S (2014). Are serum-free and xeno-free culture conditions ideal for large scale clinical grade expansion of Wharton's jelly derived mesenchymal stem cells? A comparative study. Stem Cell Res Ther.

[47] Badowski M, Muise A, Harris DT (2014). Mixed effects of long-term frozen storage on cord tissue stem cells. Cytotherapy.

[48] Romanov YA, Balashova EE, Volgina NE, Kabaeva NV, Dugina TN, Sukhikh GT (2016). Isolation of multipotent mesenchymal stromal cells from cryopreserved human umbilical cord tissue. Bull Exp Biol Med.

[49] Dulugiac M, Moldovan L, Zarnescu O (2015). Comparative studies of mesenchymal stem cells derived from different cord tissue compartments - the influence of cryopreservation and growth media. Placenta.

[50] Fong CY, Subramanian A, Biswas A, Bongso A (2016). Freezing of fresh Wharton's jelly from human umbilical cords yields high post-thaw mesenchymal stem cell numbers for cell-based therapies. J Cell Biochem.

[51] Yang Y, Melzer C, Bucan V, von der Ohe J, Otte A, Hass R (2016). Conditioned umbilical cord tissue provides a natural three-dimensional storage compartment as in vitro stem cell niche for human mesenchymal stroma/stem cells. Stem Cell Res Ther.

[52] Skiles ML, Brown KS, Tatz W, Swingle K, Brown HL (2018). Quantitative analysis of composite umbilical cord tissue health using a standardized explant approach and an assay of metabolic activity. Cytotherapy.

